# The effect of magnesium sulphate on postoperative analgesia requirements in gynecological surgeries

**DOI:** 10.4274/tjod.02439

**Published:** 2015-03-15

**Authors:** Sara Asadollah, Mansoureh Vahdat, Payman Yazdkhasti, Nasrin Nikravan

**Affiliations:** 1 Iran University Faculty of Medicine, Rasool-e-Akram Hospital, Clinic of Obstetrics and Gynecology, Tehran, Iran; 2 Iran University Faculty of Medicine, Rasool-e-Akram Hospital, Clinic of Anesthesiology and Pain, Tehran, Iran

**Keywords:** Magnesium sulphate, pethidine, anesthesia, laparotomy

## Abstract

**Objective:**

Recent studies have shown the positive effect of magnesium sulphate (MgSO4) on pain reduction and postoperative analgesic requirements in patients undergoing surgery. We assessed the effect of MgSO4 on intra-operative and postoperative analgesic requirements in patients undergoing lower abdominal gynecological laparotomy.

**Materials and Methods:**

This randomized clinical trial was conducted on 30 female patients at Rasool-e-Akram (referral and academic) hospital in Tehran from August 2012 to March 2013. The patients who were candidates for gynecologic surgeries (hysterectomy and/or myomectomy) were randomized into study (n=15) and control (n=15) groups. Same anesthetic technique was used in all patients. Besides induction of the anesthesia in the study group, we administered MgSO4 50 mg/kg/hr intravenously (IV) for analgesic purposes as a bolus dose and then 8 mg/kg IV as maintenance dose. Control group received the same anesthetic agents and the same amount of isotonic saline instead of MgSO4. Analgesic consumption was measured in both groups postoperatively within 24 hours. The visual analog scale (VAS) was used for the evaluation of postoperative pain in both groups.

**Results:**

There was a decrease in analgesic consumption and pain in the group receiving MgSO4, in comparison to control group. Pain severity assessment, 24 hours post operatively showed similar results in both groups. There was a statistically significant difference in prescribed dose of pethidine between study and control groups (p=<0.0001).

**Conclusion:**

Intra-operative MgSO4 is effective in postoperative pain control following lower abdominal laparotomy. Further studies with larger sample sizes and longer follow-up should be performed to obtain more information about safety and to determine whether doses of MgSO4 can provide postoperative analgesic benefits.

## INTRODUCTION

Postoperative pain control has always been an arguable issue amongst surgeons. The incidence of postoperative pain described by patients differs in various studies. In one of these studies, approximately 75% of patients undergoing surgery suffered from acute postoperative pain^(1)^. Appropriate analgesia can reduce morbidity and complications in surgical patients by blunting autonomic, somatic and endocrine reflexes^(2)^. Abdominal hysterectomy and myomectomy are the most common major gynecological surgeries that are performed using laparotomy method and post-operative pain control is one of the main problems in patients undergoing these operations^(3)^.

Magnesium is an intracellular cation that contributes to adjustment of enzyme reactions and adjustment of ionic channels. It is also being used for anesthetic purposes due to its blocking effect on calcium channels^(4,5)^. Although its mechanism of action is not clearly understood, interference in N-methyl-D-Aspartate (NMDA) receptor regulations is assumed to play an important role^(6)^.

Several studies have evaluated the role of magnesium sulfate (MgSO4) as an agent for pain control and reduction of analgesia requirement intra-and post-operatively. In some of these studies, researchers concluded that MgSO4 may reduce the need for opioid analgesic agents^(7,8,9,10)^.

Narcotics such as pethidine are the most widely used and cost-effective agents in postoperative pain control. However, side effects associated with their usage is a concern^(11)^. Finding a safe and cost-effective method for reducing pain has always been a debate and issue in surgical fields. The aim of this study was to evaluate the effect of MgSO4 on intra-operative and postoperative analgesic requirements in gynecologic surgeries.

## MATERIALS AND METHODS

This double-blind study was carried out on 30 women aged 36 to 56 years old who fulfilled the American Society of Anesthesiology (ASA) class I-II criteria for elective lower abdominal laparotomy (hysterectomy and myomectomy) in a tertiary academic hospital. This study was approved by the Ethics Committee of Iran University of Medical Sciences. Exclusion criteria included allergy to MgSO4, renal or hepatic or cardiovascular dysfunction, neurological disorders, atrioventricular conduction disturbance, opioid or analgesic abuse, and long-term treatment with calcium channel blockers or magnesium. Prior to induction, monitoring was started with electrocardiogram (ECG), pulse oximetry and noninvasive blood pressure measurement.

In the operating room, isotonic saline infusion (normal saline) was started and continued during the operation for all patients.

Technique of general anesthesia was similar in all patients: midazolam (0.2 mg/kg) as premedication, propofol (2 mg/kg), fentanyl (2 µg/kg) as induction agents, and atracorium besylate (0.5 mg/kg) for tracheal intubation. In the maintenance phase, propofol (100-150 mg/kg/min) and remifentanyl (0.4 µg/kg/min) were used. Fentanyl (1 mg/kg) was administered before the end of operation, and at the end of surgery, neostigmine (0.04 mg/kg) and atropine (0.02 mg/kg) were given in order to reverse the action^(12)^.

A total of 30 patients were randomized to receive 50 mg/kg of MgSO4 in 100 ml of isotonic saline over 10 min immediately before the induction of anesthesia and then 8 mg/kg/hr by continuous IV infusion until the end of the operation (study group) or to receive the same volume of an isotonic saline solution (control group). The patients and anesthesiologist were blinded. The study data were recorded by an observer who was blinded to both groups. MgSO4 and anesthetic agent infusions were discontinued at the time of skin closing. After the end of surgery, the visual analog scale (VAS) from 0 to 10 (with 0 representing no pain and 10 representing the worst imaginable pain) was used for postoperative pain evaluation several times (30 min, 4 hr, 12 hr and 24 hr after the operation).

If a patient complained of pain or VAS was higher than 4, then 30 mg of pethidine was injected and, if needed, the same dose was repeated q 3 hours till the patient was free of pain. The total dose and number of doses injected were recorded. Postoperative nausea, vomiting, respiratory rate, blood pressure, heart rate, deep tendon reflex, and level of consciousness were recorded throughout the study period.

### Statistical Analysis

Data were registered in checklists including demographic characteristics, patient’s complaints, VAS score, and amount of pethidine dosage. Numerical variables were reported as mean ± standard deviation (SD). We used an independent-t-test and chi-square test to compare quantitative and qualitative variables, respectively. A p value of ≤0.05 was considered statistically significant. All analyses were performed using SPSS for Windows (SPSS version 20, Chicago, Illinois, USA).

## RESULTS

**Figure 1:**
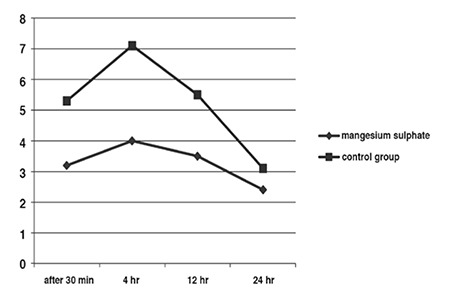
Pain score after operation in two groups

The mean age of patients was 48.85±4.8 years (range: 36-56) (Table 1). The patients in control group showed higher postoperative pain on average on VAS in comparison with study group at the end of 30 min, 4 hours and 12 hours. Evaluation of pain yielded similar results in both groups, except in first 12 hours. Pain scores were significantly lower in the first 12 hours in patients receiving MgSO4 (p=<0.0001) (Figure 1).

Incidence of nausea or vomiting and cause of surgery (abdominal hysterectomy or myomectomy) were similar in both groups (p=0.3, p=0.9, respectively).

## DISCUSSION

In this randomized clinical trial, the effect of MgSO4 on intra-and postoperative analgesic requirements in gynecologic surgeries was assessed. We have shown in this study that MgSO4 infusion during operation reduced postoperative pain (Figure 1).

In this study, we prescribed 50 mg/kg MgSO4 as a bolus dose and 8 mg/kg/hr as a maintenance dose. Serum Mg concentrations in study group was higher than in patients in control group after surgery, but there were no complications associated with hypermagnesaemia.

Based on our results, opioid consumption and pain severity scores were both significantly lower in the study group at 30 minutes, 2 hours, 4 hours and 12 hours, postoperatively.

Previous studies have also reported that magnesium administration significantly reduces opioid analgesic drug requirements^(13,14,15)^.

Also it has been shown that MgSO4 may reduce anesthetics requirements. In their study, Choi and colleagues demonstrated that the usage of propofol reduced from 167 to 81 mg/kg/min after administering bolus dose of MgSO4 (50 mg/kg) followed by continuous MgSO4 infusion (8 mg/kg/min) in gynecological surgery^(14)^.

Seyhan and colleagues compared the effect of MgSO4 infusion on amount of anesthetic requirements and postoperative pain. Based on the results of their study, infusion of MgSO4 in patients significantly reduced the amount of intra-operative propofol and neuromuscular blocking agent. Postoperative pain and opioid analgesics consumption was also reduced^(16)^.

On the other hand, some studies have shown that MgSO4 does not reduce postoperative analgesic requirements. For example, KO et al. showed that cerebrospinal fluid (CSF) concentration of magnesium did not increase after IV administration of MgSO4 and it had no effects on postoperative pain. On the other hand, they reported an inverse relation between cumulative postoperative analgesic consumption and the CSF magnesium concentration. They concluded that CSF magnesium concentration affects postoperative pain, but perioperative IV magnesium administration had no analgesic effects^(17)^. Ryu J H and colleagues showed that MgSO4 infusion intra-operatively did not reduce propofol requirements^(18)^.

Anesthetic effect of magnesium is believed to be due to magnesium and calcium ions competition in pre-synaptic calcium channels^(15)^. However, the mechanism of analgesic effect of magnesium is not completely understood, but it has been proposed that MgSO4 can prevent central sensitization after peripheral nociceptive stimulation and suppress hypersensitivity, and this analgesic property is due to its action on NMDA receptors and calcium channels^(18)^. Previous studies have also proved this finding that one of the main target sites for general anesthesia is release of glutamate by pre-synaptic calcium channels^(19,20,21)^.

Non-steroidal anti-inflammatory drugs (NSAIDs) are used as an analgesic option for postoperative pain, however, many concerns about adverse effects of these drugs, such as gastrointestinal bleeding, acute renal failure and allergic reactions have limited their usage^(22)^. As shown in our study, intra-operative MgSO4 is effective in postoperative pain and it reduces analgesic requirements, therefore, the need to consume NSAIDs postoperatively may be also decreased.

Our study had some limitations which should be noted; we should have determined concentration of magnesium before the operation and not only after operation, thus, we could evaluate the relationship between magnesium concentration and pain severity following the operation. Also the sample size of our study was small and another study with a larger sample size is necessary to be conducted.

## CONCLUSION

Intra-operative and continuous infusion of MgSO4 to women who have undergone lower abdominal laparotomy significantly reduced opioid requirements and pain postoperatively. Further studies with larger sample sizes and longer follow-up should be performed to obtain more information about safety and to determine whether doses of MgSO4 can provide postoperative analgesic benefits.

## Figures and Tables

**Table 1:**
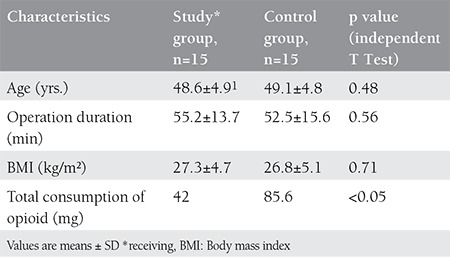
Demographic data, amount of analgesic usage and operation time
